# Verbal Cue Use and Domains of Pre-Competitive Confidence in Competitive Weightlifters

**DOI:** 10.3390/sports14100434

**Published:** 2026-10-06

**Authors:** Marianne Huebner, Nicholas Mortensen, Corinne T. Zimmerman, Wenjuan Ma, Nicholas D. Myers

**Affiliations:** 1 Department of Statistics and Probability, Michigan State University, East Lansing, MI 48864, USA; 2 Department of Kinesiology, Michigan State University, East Lansing, MI 48864, USA; morten45@msu.edu (N.M.); myersni1@msu.edu (N.D.M.); 3 Department of Sport Leadership and Management, Miami University, Oxford, OH 45056, USA; zimmerc6@miamioh.edu; 4 Center for Statistical Training and Consulting, East Lansing, MI 48864, USA; mawenjua@msu.edu

**Keywords:** attention cues, self-efficacy, sport confidence, weightlifting, pre-competition appraisal

## Abstract

Competitive weightlifting requires athletes to perform complex technical movements under high physical and psychological demands. This study aimed to characterize pre-competition verbal cue use among competitive weightlifters, examine the structure of pre-competition confidence, and explore associations between cue categories and confidence domains. Athletes competing at the 2025 USA Weightlifting American Finals completed a survey prior to the start of the competition assessing verbal cues used and confidence in performance goals, mental resilience, and physical readiness. Verbal cues were categorized as control, neutral, internal, external, or promotion-focused. Exploratory and confirmatory factor analyses supported a three-factor structure of confidence comprising performance goals, mental resilience, and physical readiness. Among 122 athletes who reported using verbal cues, external (39%) and control (35%) cues were most common, and more than half reported using multiple cue types. Women more frequently reported promotion-focused cues and less frequently reported control cues than men. Exploratory structural regression analyses indicated that control cues were positively associated with mental resilience and physical readiness, while promotion-focused cues were positively associated with physical readiness. These findings provide field-based descriptive evidence regarding verbal cue use among competitive weightlifters and suggest that different cue categories may be related to distinct dimensions of pre-competition confidence.

## 1. Introduction

In weightlifting competitions, athletes aim to lift the heaviest weights in their weight category during three attempts for the snatch and three attempts for the clean and jerk. Successful performance requires not only strength, speed, and technical precision [1,2], but also the ability to execute complex motor skills under time pressure and competitive stress [3]. Therefore, psychological factors such as confidence, attention, and pre-competition preparation may play an important role in competitive outcomes.

Confidence, often framed as self-efficacy or sport confidence, refers to an athlete’s belief in their ability to perform well or to succeed [4,5]. Vealey’s model of sport confidence emphasizes that confidence is multidimensional, drawing on different sources and manifesting in different constructs depending on task demands [6,7]. In weightlifting, these constructs may include confidence in achieving performance goals (e.g., planned attempts), confidence in physical and technical readiness, and confidence in psychological resilience following failed attempts. Confidence may be a useful factor of precompetitive appraisal, or an athlete’s perceived ability to cope with the demands of an upcoming competition [8,9].

Athletes employ a range of psychological strategies during the pre-competition period to regulate attention and confidence, including the use of verbal attentional cues and self-talk [10,11,12,13,14]. Domain-specific attentional cues are categorized as internal (directed toward body movements or sensations, e.g., “elbows up”, “strong legs”) or external (directed toward the effects of movement on the environment, e.g., push through the legs”, “keep the bar close”), with a substantial body of research suggesting performance benefits of an external focus of attention [11,15,16]. In addition to internal and external cues, athletes may use domain general control or neutral cues that are not tied to specific movement mechanics (e.g., “stabilize”, “take a breath”), as well as motivational cues intended to regulate arousal or reinforce goals. Motivational cues can emphasize accomplishments such as aspirations (“promotion focused”) or safety (“prevention focused”) [17]. Emerging evidence suggests that athletes may combine multiple cue types depending on situational demands. Weightlifting competitions present unique constraints, including limited preparation time before each attempt and irreversible load selections, which may influence both the form and function of attentional cues. However, much of this work has been conducted in laboratory or training settings, often with small samples or novice athletes, and may not generalize to competition contexts or to highly trained performers [11,14,18]. Weightlifting competitions involve time pressure, public performance, strategic attempt selection, and the possibility of immediate failure. Consequently, the cueing strategies used by athletes in competition may differ from those observed in experimental settings. Understanding how experienced athletes naturally use verbal cues during competition may therefore provide valuable information for both coaches and researchers.

Despite growing interest in attentional focus and self-talk strategies, there is limited empirical evidence describing the types of cues weightlifters use in actual competition or how these cues relate to different aspects of pre-competition confidence [11,14,19]. Moreover, potential sex differences in cue use and confidence appraisals remain underexplored in this sport. Addressing these gaps is important for informing applied practice in strength-based sports.

The purpose of this study was to 1. describe the types of pre-competition verbal attentional cues used by competitive weightlifters, 2. examine the dimensional structure of pre-competition confidence in this population, and 3. evaluate associations between cue categories and distinct confidence domains. A secondary aim was to explore sex differences in cue use.

## 2. Materials and Methods

### 2.1. Study Population

**Figure 1:**
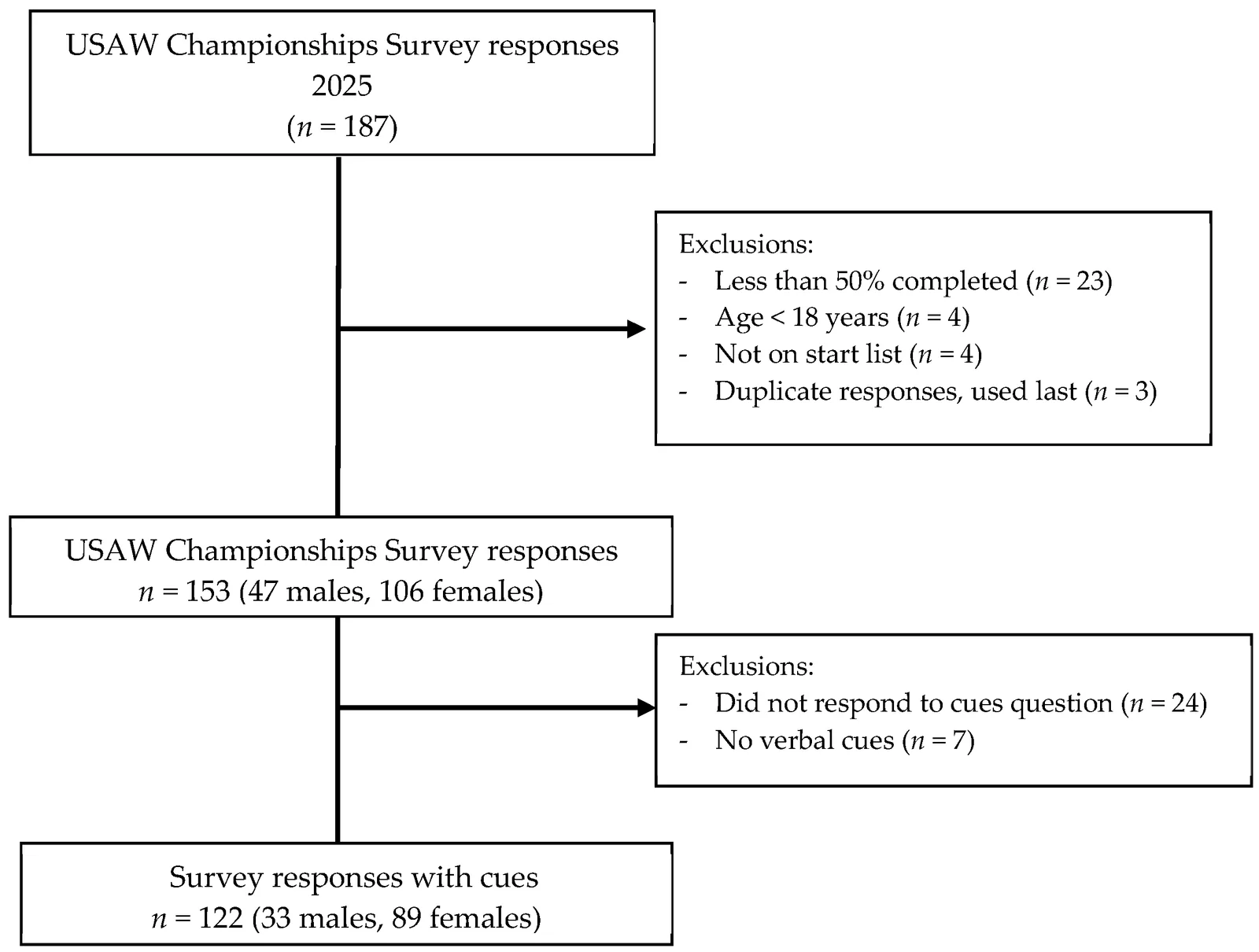
STROBE flow diagram [20].

Competitive weightlifters competing at the 2025 USA Weightlifting American Finals were invited to participate. Registered athletes were invited to participate in the study via USAW’s email system with 2 reminders at 3, 2, and 1 week before the start of the competition and were asked to complete the survey before the start of their competition session. Flyers were posted at the registration desk, weigh-in rooms, and in the training hall. Participants were asked to fill out the survey prior to their session. The survey was administered online via Qualtrics (Provo, UT, USA). Data collection began on 16 November and finished on 7 December 2025. Of 187 respondents, exclusions due to incomplete responses, age < 18, duplicate entries, or absence from the start list resulted in a final analytic sample of 122 athletes who reported using verbal cues (Figure 1).

### 2.2. Measures

Demographic and training variables included age, years of competitive experience, weekly training volume, and coach involvement.

Verbal cues were collected using an open-ended survey question: “Please share any verbal cues you or your coach use that help you feel ready to lift.” Athletes could report one or more cues that they typically use during competition preparation. These were then categorized as control, neutral, internal, external, or promotion-focused. Categories were defined a priori using a coding manual developed from attentional focus and motivational cue literature. Two investigators (MH and NM) independently coded responses using a predefined manual and reached agreement following consensus review. A summary of coding categories is provided in Table 1, and the full coding manual and examples are publicly available on the Open Science Framework (OSF, https://osf.io/gakwz).

Participants rated pre-competition confidence across 11 items assessing confidence in goal attainment, mental resilience, and physical and technical readiness using a five-point Likert scale (very low to complete confidence). The complete survey instrument is available on OSF (https://osf.io/gakwz). Information about goals for the competitions in snatch, clean and jerk, and total, and whether these goals were personal records or new national records was also collected. Actual performances from the competition results were available for comparison. Success was defined as the percentage of the best attempt achieved in relation to the competition goal.

### 2.3. Statistical Analysis

Descriptive statistics were computed using counts and percentages. Sex-stratified descriptive analyses were prespecified because previous sport psychology and weightlifting studies have reported potential sex differences in confidence, motivation, coaching interactions, and competition-related psychological factors. Because the dimensionality of pre-competition confidence had not previously been established in competitive weightlifters, exploratory factor analysis (EFA) was used to investigate the underlying factor structure, followed by confirmatory factor analysis (CFA) to evaluate the resulting measurement model. Structural regression models were then estimated by incorporating the five cue-related variables as predictors of the latent confidence factors, thereby allowing the examination of the hypothesized relationships while accounting for measurement error. This sequential approach follows recommended structural equation modeling procedures by evaluating the measurement model prior to examining the structural relationships among constructs [21,22]. Because the EFA and CFA were conducted using the same sample, the CFA should be interpreted as providing support for the plausibility of the factor structure rather than as an independent validation. Replication in an independent sample is needed to confirm the stability of the factor solution. Accordingly, associations between cue categories and confidence domains should be considered exploratory and hypothesis-generating. Relationships between confidence ratings for the snatch, clean and jerk, and total goals and subsequent performance relative to those goals were evaluated using Spearman correlation coefficients. This nonparametric approach was selected because confidence ratings were measured on ordinal Likert scales.

#### 2.3.1. Exploratory Factor Analysis (EFA)

To determine the latent structure of the confidence items, an EFA was performed on the polychoric correlation matrix. Two-, three-, and four-factor solutions were examined. Candidate solutions were compared using model fit indices (comparative fit index CFI, Tucker–Lewis index TLI, root mean square error of approximation RMSEA, and standardized root mean square residual SRMR), examination of factor loading patterns and cross-loadings, and substantive interpretability of the resulting factors. For interpretation of the retained factor solution, loadings ≥ |0.40| were considered primary loadings, whereas loadings ≥ |0.30| on an additional factor were considered meaningful cross-loadings. Because the dimensional structure of pre-competition confidence in competitive weightlifters had not been established previously, emphasis was placed on identifying a parsimonious solution that produced conceptually coherent and practically meaningful confidence domains. The final factor solution was selected based on model fit, factor loading patterns, substantive interpretability, and parsimony. Detailed model comparisons are provided in the supplement. Models were estimated with a geomin rotation and the Unweighted least squares with mean- and variance-adjusted (ULSMV) estimator. Parallel analysis was conducted as an additional factor-retention criterion [23,24].

#### 2.3.2. Confirmatory Factor Analysis (CFA)

Confidence items were treated as ordered categorical indicators and analyzed using the weighted least squares mean- and variance-adjusted estimator (WLSMV). Candidate factor solutions identified through the EFA were subsequently evaluated using CFA. Model fit was evaluated with the scaled chi-square statistic, CFI, TLI, RMSEA with 90% confidence intervals, and SRMR. Consistent with commonly used structural equation modeling guidelines, CFI and TLI values ≥ 0.90 and RMSEA and SRMR values ≤ 0.08 were considered indicative of acceptable fit. However, model fit was evaluated using all indices collectively rather than relying on strict cutoff criteria [25]. Reliability of each factor was estimated using McDonald’s omega total [26].

#### 2.3.3. Structural Regression Models

Structural regression models were used to examine associations between cue categories and latent confidence constructs [21,22,25,26]. Because athletes could report multiple cue types, cue categories were coded as non-mutually exclusive indicators. All cue categories were entered simultaneously as exogeneous predictors into the structural regression model to estimate their associations with confidence domains while adjusting for the presence of the other cue categories. The models were estimated using the same weighted least squares mean- and variance-adjusted (WLSMV) estimator and ordinal specification of confidence indicators used in the CFA. All direct paths from the cue categories to the latent confidence factors were specified, and factor covariances were freely estimated. Model fit was evaluated using the same indices as for the CFA (scaled χ^2^, CFI, TLI, RMSEA, and SRMR). Regression coefficients (*B* and standardized *β*), standard errors, 95% confidence intervals, *p*-values, and the proportion of variance explained (*R^2^*) for each latent factor were reported.

Missing values for participant characteristics are reported in Table 2. Analyses of participant characteristics were conducted using available data. The 11 confidence items used in the EFA, CFA, and structural regression analyses contained no missing responses among the 122 athletes included in the analytic sample.

Statistical analyses were performed using the statistical software R v.4.6.1. For the factor analysis and regression models, the lavaan package (v.0.7.2) [27] and the semTools package (v.0.5.9) [28] were used. Because the confidence items were ordinal, all models were estimated with robust weighted least squares (estimator = “WLSMV” for CFA or “ULSMV” for EFA) and the theta parameterization. The study was reported according to STROBE guidelines [20].

## 3. Results

### 3.1. Participant Characteristics

Table 2 lists characteristics or 122 participants who provided verbal cues with median age 40 years (range 18–72 years). Weightlifting competition experience was more than 3 years in 71.3% of the participants. More women utilize coaches compared to men for training (98.9% and 79.8%, respectively) and for the competition (96.6% and 75.8%, respectively). Overall, 69.0% respondents intended to set personal records and 9.5% national records.

### 3.2. Verbal Cue Use

Of the 153 eligible respondents, 122 provided verbal cues. No statistically significant differences were observed between athletes who provided verbal cues and those who did not with respect to age, sex, years of competition experience, training volume, or coaching support.

**Figure 2:**
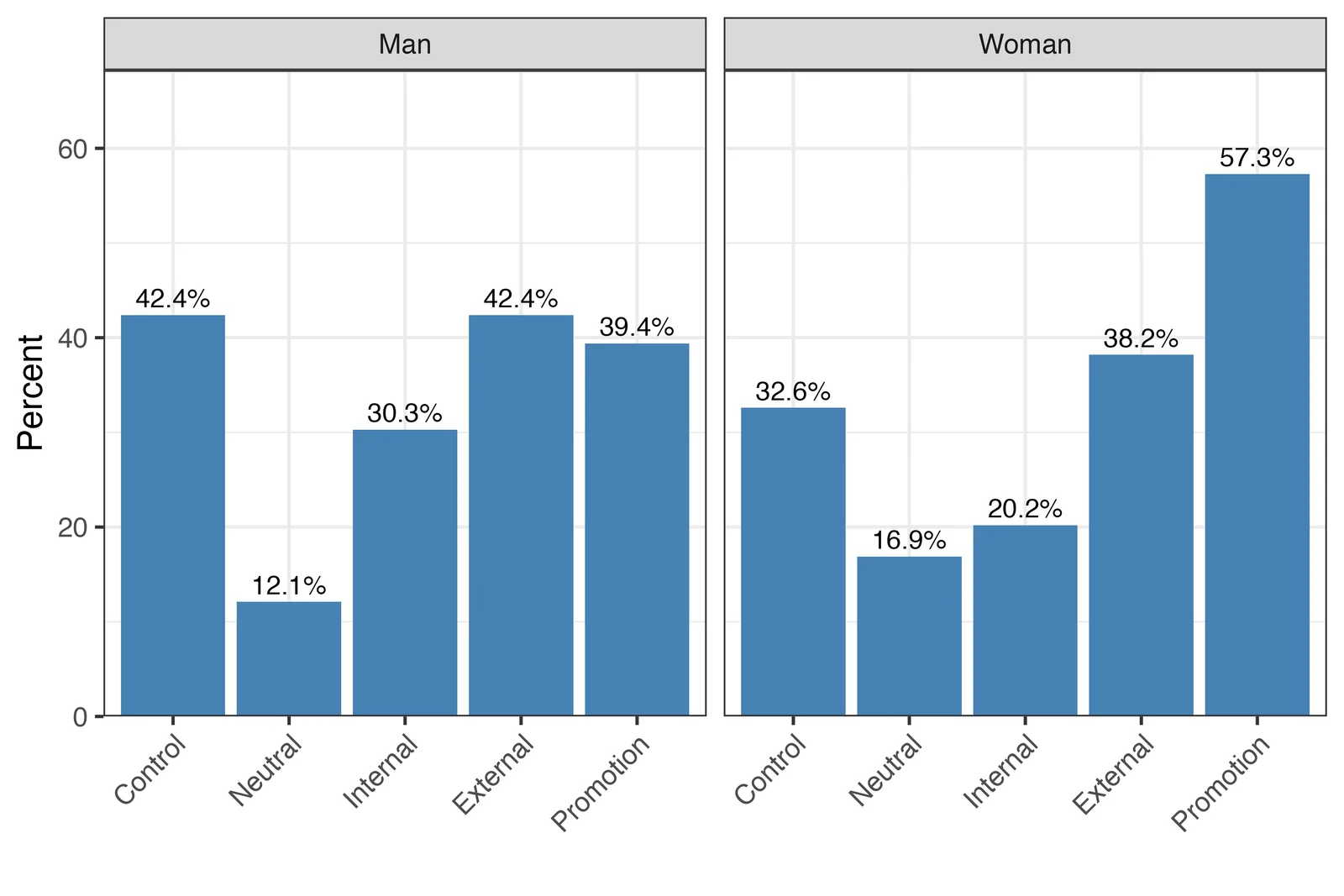
Distribution of pre-competition attentional cue categories reported by competitive weightlifters, stratified by sex. Values represent the percentage of athletes endorsing each cue category. Athletes could report multiple cue types.

Almost half of the cues used one or two words; most were less than four words. More than half of the participants rely on two or more types of cues (54.1%). Women used more promotion cues than men (57.3% vs. 39.4%) and fewer control cues (32.6% vs. 42.4%) (Table 3, Figure 2). The most frequent body parts in the cues were legs (35.9%), feet (20.3%), or arms (15.6%).

### 3.3. Pre-Competition Confidence

**Figure 3:**
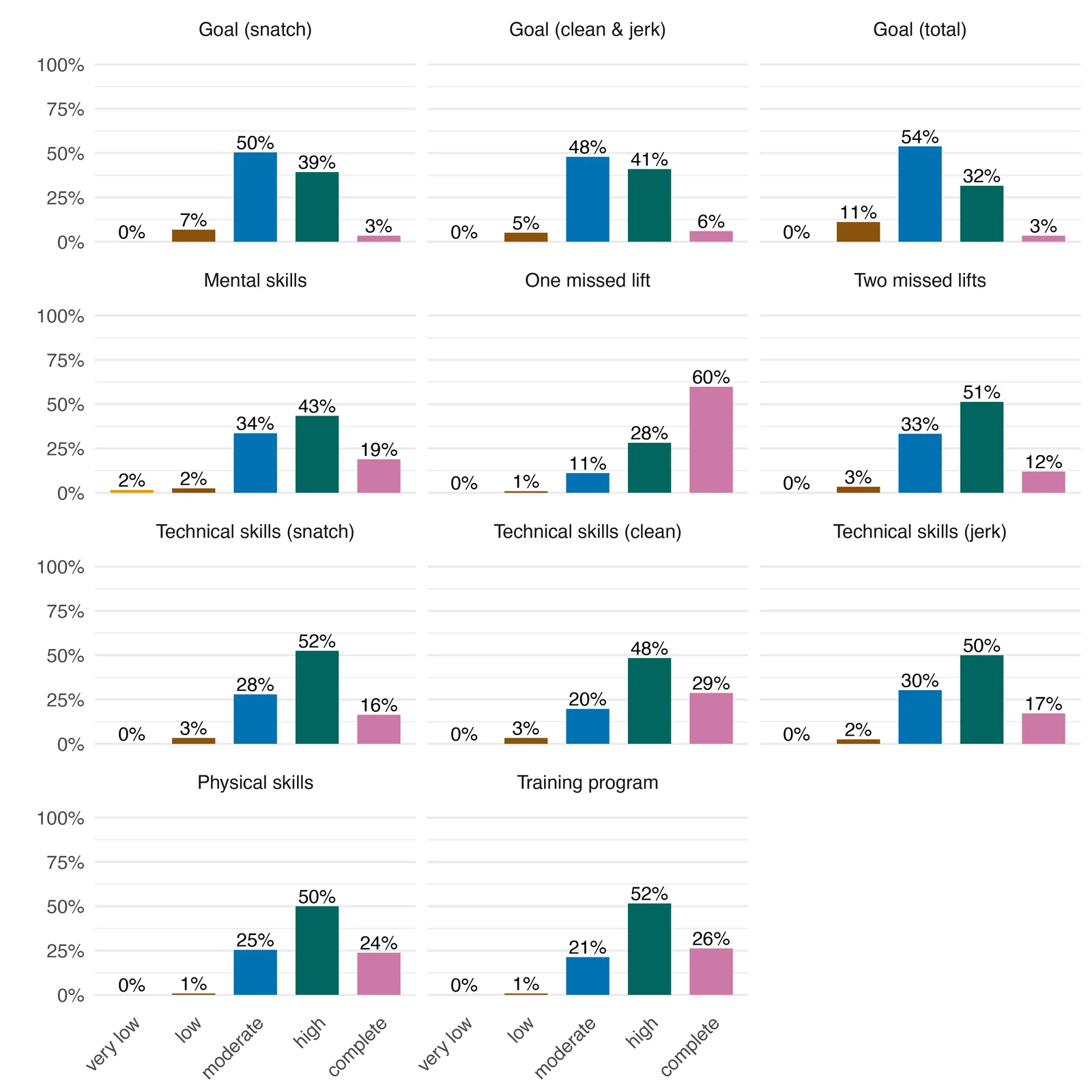
Distribution of pre-competition confidence ratings across assessed confidence domains among competitive weightlifters. Bars indicate the proportion of athletes reporting each confidence level (very low to complete confidence) for each domain.

The confidence in the training program for the competition preparation was high, 51.6% reported high confidence and 26.2% complete confidence (Table 4, Figure 3). Confidence in the technical skills for the clean (28.7% complete confidence) was higher than for snatch and jerk (16.4 and 17.2%, respectively). Self-confidence in achieving the personal goals for snatch, clean and jerk, and total were not correlated with the ratio of actual weight lifted to the personal goals (*r* = 0.15 to 0.17).

### 3.4. Factor Analysis

Exploratory factor analysis supported a three-factor structure representing confidence in performance goals, mental resilience, and Physical Competition Readiness (Table 5). Two-, three-, and four-factor solutions were evaluated. Although the four-factor solution demonstrated the best statistical fit, improvements in fit relative to the three-factor solution were modest. The three-factor solution was retained because it provided a more parsimonious and conceptually coherent representation of confidence in competitive weightlifting while maintaining acceptable model fit. Detailed results for all candidate models are presented in the Supplementary Materials (Table S1). The retained three-factor solution demonstrated acceptable fit (CFI = 0.992, TLI = 0.984, RMSEA = 0.044, SRMR = 0.037). Internal consistency was acceptable for all three factors. Confirmatory factor analysis provided additional support for the retained three-factor structure (CFI = 0.989, TLI = 0.986, RMSEA = 0.058, SRMR = 0.057; Table S2 in the Supplementary Materials). Internal consistency was acceptable for all three factors with ω total values of 0.860 for Goals, 0.793 for Mental Resilience, and 0.823 for Physical Competition Readiness. The latent confidence domains were moderately correlated, supporting the use of an oblique factor solution (Table S3). The factor labels were assigned after examination of the loading patterns and item content and were not specified a priori. Factor 1 was defined by the three goal-attainment items and was interpreted as Goals. Factor 2 was primarily defined by confidence in performing after one or two missed lifts, together with confidence in mental skills, and was interpreted as Mental Resilience. The strongest loadings on this factor reflected confidence in performing successfully after missed lifts, while confidence in mental skills also contributed to the construct, supporting its interpretation as confidence in coping with setbacks and maintaining performance under competitive pressure (Table 5). Factor 3 was defined by confidence in physical skills, technical execution, and training preparation and was interpreted as Physical Competition Readiness. Although the training program item demonstrated similar loadings on Mental Resilience (0.436) and Physical Competition Readiness (0.428), it was assigned to Physical Competition Readiness because its content relates most directly to preparation for competition. The observed cross-loading suggests that confidence in competition preparation may contribute to both perceptions of physical readiness and confidence in coping with competitive challenges.

### 3.5. Associations Between Cue Categories and Confidence Factors

Structural regression was used to examine associations between the five pre-competition attentional cue categories and the three latent confidence factors (Table 6). All cue categories were entered simultaneously, so each coefficient represents the association of that cue category after accounting for the others. The model demonstrated acceptable fit: χ^2^ (81) = 101.60, *p* = 0.061; CFI = 0.987; TLI = 0.991; RMSEA = 0.045 (90% CI [0.000, 0.071]); SRMR = 0.063. The cue categories explained 8.5% of the variance in Goals, 6.6% in Mental Resilience, and 8.2% in Physical Competition Readiness.

Control cues were positively associated with Mental Resilience (*B* = 0.455, 95% CI [0.014, 0.896], *β* = 0.206, *p* = 0.043) and Physical Competition Readiness (*B* = 0.492, 95% CI [0.033, 0.950], *β* = 0.221, *p* = 0.036). Promotion-focused cues were positively associated with Physical Competition Readiness (*B* = 0.435, 95% CI [0.003, 0.868], *β* = 0.209, *p* = 0.049). External cues showed a marginally significant positive association with Goals (*B* = 0.406, 95% CI [−0.034, 0.845], *β* = 0.188, *p* = 0.071). No other paths reached statistical significance; all corresponding confidence intervals included zero.

Because the model estimated 15 structural paths in a sample of *n* = 122, the resulting paths should be interpreted cautiously. Confidence intervals for many paths were relatively wide, reflecting uncertainty in individual parameter estimates. Consequently, the structural regression should be considered exploratory and hypothesis-generating rather than confirmatory.

## 4. Discussion

The purpose of this study was to examine pre-competition attentional cue use among competitive weightlifters and to evaluate whether different cue types were associated with distinct dimensions of pre-competition confidence. The main findings indicate that weightlifters use a variety of brief verbal cues during competition, that pre-competition confidence is represented by three related but distinguishable domains (confidence in achieving performance goals, confidence in mental resilience, and confidence in Physical Competition Readiness), and that specific cue categories are differentially associated with confidence in mental resilience and Physical Competition Readiness.

### 4.1. Prevalence of Cues: External, Internal, General Domain, or Promotion

Athletes frequently reported using short cues of one to four words (80%), while half of them mentioned one- or two-word cues. This is consistent with the temporal constraints of competitive weightlifting, where lifters have limited time to initiate an attempt after being called to the platform [1,2]. The adoption of a single focus may be most beneficial [11]. Giving too many instructions can be detrimental to performance [17].

External and control cues were the most prevalent across the sample, and more than half of the athletes reported using multiple cue types. This suggests that athletes may use combinations of attentional and motivational strategies rather than relying on a single cueing approach. Consequently, associations observed for individual cue categories should be interpreted within the broader context of athletes’ overall cueing repertoires rather than as isolated strategies. However, pairwise correlations among cue categories were small (all |*r*| ≤ 0.27), suggesting limited overlap among the categories. This pattern suggests that experienced weightlifters may employ flexible attentional strategies tailored to the demands of competition. This is aligned with previous findings that experienced athletes may utilize multiple attentional strategies for their performances [29]. The prevalence of multiple cue use suggests that experimentally examining a single cue type may not fully capture how athletes prepare for competition in real-world settings. Future studies should consider both individual cue categories and combinations of cues when investigating relationships with confidence and performance.

There is a body of literature supporting an external focus of attention for performance outcomes [11,15,16,30]. However, in highly skilled athletes, a change in focus could lead to decreased performance [13]. To minimize this potential performance decrements, some authors suggest using neutral or control cues (‘holistic’) may be preferable [19,31]. Such cues may help preserve automatic motor control processes while maintaining composure and balance.

### 4.2. Structure of Pre-Competition Confidence

Exploratory and confirmatory factor analyses supported a three-factor structure of pre-competition confidence, comprising confidence in achieving performance goals, confidence in mental resilience, and confidence in Physical Competition Readiness. The mental resilience domain was largely defined by confidence in recovering from missed lifts, which was interpreted as a manifestation of competition-specific psychological resilience. These domains map closely onto key components of Vealey’s model of sport confidence [6,7]. The distinction between confidence in performance goals, resilience following missed lifts, and physical readiness may be particularly relevant in weightlifting because competition success depends not only on physical capability but also on an athlete’s ability to recover psychologically following unsuccessful attempts. Unlike many sports, missed lifts have immediate consequences for subsequent attempt selection and may alter competitive standing. Consequently, confidence in recovering from setbacks may represent a unique aspect of competition-related confidence in weightlifting. The moderate correlations among confidence factors indicate that these domains represent distinct aspects of pre-competition appraisal. This finding supports the importance of assessing confidence multidimensionally, particularly in a technically demanding individual sport such as weightlifting.

### 4.3. Associations Between Cues and Confidence Domains

In our study, control cues were positively associated with confidence in mental resilience and Physical Competition Readiness, while promotion-focused cues were positively associated with Physical Competition Readiness. These findings suggest that cues emphasizing composure, stabilization, or execution control may be associated with athletes’ confidence in their ability to cope with pressure and execute lifts effectively. These associations should be interpreted as exploratory and require confirmation in independent samples.

Control cues may support executive control processes, such as attentional regulation and emotional inhibition, which are important for maintaining technical consistency or for recovery following failed attempts [32]. For example, “pause” and “stabilize” refer to aspect of the lift to balance after the catching the bar overhead in a squat or split position and getting ready to stand up, while a control cue “finish the pull” refers to the timing of the triple extension before turning over the bar to catch overhead. Previous studies noted that control or neutral cues that do not focus on specific steps of a movement resulted in better performance in sprint or throws in highly trained athletes [13,18]. Promotion-focused cues, which emphasize success or goal attainment, were positively associated with higher levels of perceived readiness. These cues may reflect positive expectations related to strength and technical competence, although the direction of this relationship cannot be determined from the present study.

External cues demonstrated a positive, though non-significant, association with confidence in achieving performance goals. This pattern is consistent with the idea that external cues primarily facilitate movement execution [11,16,19], whereas confidence in goal attainment may be shaped more strongly by prior performance experiences, training history, and contextual factors [4,7,33].

### 4.4. Sex Differences

Women more frequently reported promotion-focused cues and less frequently reported control cues compared to men. These differences may reflect variation in coaching communication or competitive experiences between sexes [34]. However, these findings are descriptive and should be interpreted cautiously because the study was not designed or powered to evaluate sex-specific mechanisms. Future research should examine whether differences in coaching practices, competitive experiences, or other factors contribute to cue selection.

### 4.5. Strengths and Limitations

A key strength of this study is the inclusion of a large and diverse sample of experienced weightlifters competing at a high-stakes national event. The examination of attentional cues in real-world competition addresses a limitation of prior research, which has primarily focused on laboratory or training contexts [11,14,16,19].

Several limitations should be considered. First, confidence and cue use were assessed using self-report measures and may be subject to reporting bias. Second, the confidence-factor structure should be interpreted cautiously because both EFA and CFA were conducted using the same sample. Although the retained three-factor solution demonstrated acceptable fit and was supported by multiple robustness checks, independent validation in a larger sample is needed to confirm the stability and generalizability of the factor structure. Third, the cue question assessed athletes’ general cueing strategies used to feel ready to lift rather than cues used during a specific competition attempt, and cue use may vary across lifts and competition contexts. Because athletes could report multiple cue types, cue categories were not mutually exclusive. Pairwise correlations among cue categories were small, suggesting limited overlap among the individual cue categories. However, the present analyses did not evaluate specific cue combinations or interaction effects. Finally, cue use and confidence were measured contemporaneously; therefore, the direction of the observed associations cannot be determined. It is possible that cueing strategies are associated with greater confidence, but it is equally plausible that more confident athletes preferentially adopt certain cue types. Accordingly, the structural regression findings should be interpreted as associative rather than causal. Although analyses were restricted to athletes who reported verbal cues, comparisons of available demographic and training characteristics revealed no evidence of systematic differences between athletes who did and did not provide cue information.

## 5. Conclusions

Athletes reported using brief verbal cues used to prepare for competition attempts, often combining multiple cue types. Coaches and athletes may find it useful to discuss cueing strategies that fit the temporal constraints of a competition as part of their training and competition preparation. The present findings suggest that cue categories may be associated with different dimensions of confidence. Control-oriented cues (e.g., emphasizing stability, timing, or composure) and promotion-focused cues were positively associated with confidence in physical readiness and mental resilience during competition. Because cue use and confidence were measured contemporaneously, it is unclear whether these cues contribute to confidence, whether more confident athletes are more likely to adopt such cues, or whether both are influenced by other factors. Experimental studies are needed before recommending specific cue types to enhance confidence or performance.

## Figures and Tables

**Table 1:** Verbal cue categories and definitions used for classifying athlete-reported pre-competition cue.

Cues Category	Definition
Control	Emphasizing smooth, deliberate execution rather than rushing or jerking the movement
Neutral	Keeping the body in a safe, balanced position
External	Directing attention to the effect of the movement on the environment or an external object
Internal	Directing attention to a body part or internal sensation during movement
Promotion-focused	Encouraging striving for gains, success, and achievement.

**Table 2:** Participant characteristics of competitive weightlifters who reported pre-competition verbal cues, stratified by sex.

	Man *n* = 33	Woman *n* = 89	Combined *n* = 122
Age, median (quartiles)	44 (36, 53)	39 (32, 47)	40 (33, 50)
Start age in weightlifting, % (*n*)			
10–14 years	9.1 (3)	5.6 (5)	6.6 (8)
15–19 years	15.2 (5)	9.0(8)	10.7 (13)
20–24 years	12.1 (4)	18.0 (16)	16.4 (20)
25–29 years	27.3 (9)	10.1 (9)	14.8 (18)
30 or older	36.4 (12)	57.3 (51)	51.6 (63)
Years of competition experience			
1–3 years	21.4 (6)	31.0 (27)	28.7 (33)
4–5 years	28.8 (8)	12.6 (11)	16.5 (19)
6–9 years	32.1 (9)	34.5 (30)	33.9 (39)
10 or more years	17.9 (5)	21.8(19)	20.9 (24)
missing	5	2	7
Hours of training per week, median (quartiles)	8 (6, 10)	8 (6, 10)	8 (6, 10)
Number of training days per week, median (quartiles)	4 (2, 7)	4 (3, 6)	4 (2, 7)
Coach for training			
No coach	21.2 (7)	1.1 (1)	6.6 (8)
Coach for occasional advice	9.1 (3)	3.4 (3)	5.0 (6)
Coach for program	3.0 (1)	5.7 (5)	5.0 (6)
Coach for program and training	66.7 (22)	89.8 (79)	83.5 (101)
missing	0	1	1
Coach for competition			
No coach	24.2 (8)	3.4 (3)	9.0 (11)
Coach competition prep	57.6 (19)	89.9 (80)	81.1 (99)
Team member	(4)	3.4 (3)	5.7 (7)
Other coach	6.1 (2)	3.4 (3)	4.1 (5)
Personal record goal (PR), *n* = 116	61.3 (19)	71.8 (61)	69.0 (80)
National record goal (NR), *n* = 116	22.6 (7)	4.7 (4)	9.5 (11)

**Table 3:** Frequency of pre-competition attentional cue categories reported by competitive weightlifters, stratified by sex, % (*n*).

	Man *n* = 33	Woman *n* = 89	Combined *n* = 122
General Domain	48.5 (16)	41.6 (37)	43.4 (53)
Control	42.4 (14)	32.6 (29)	35.2 (43)
Neutral	12.1 (4)	16.9 (15)	15.6 (19)
Specific Domain	57.6 (19)	51.7 (46)	53.3 (65)
External	42.4 (14)	38.2 (34)	39.3 (48)
Internal	30.3 (10)	20.2 (18)	23.0 (28)
Motivation (promotion-focused)	39.4 (13)	57.3 (51)	52.5 (64)

**Table 4:** Distribution of pre-competition confidence ratings across confidence domains, stratified by sex.

	Man *n* = 33	Woman *n* = 89	Combined *n* = 122
Confidence in training program			
Very low confidence	0.0 (0)	0.0 (0)	0.0 (0)
Low confidence	0.0 (0)	1.1 (1)	0.8 (1)
Moderate confidence	18.2 (6)	22.5 (20)	21.3 (26)
High confidence	51.5 (17)	51.7 (46)	51.6 (63)
Complete confidence	30.3 (10)	24.7 (22)	26.2 (32)
Confidence in physical skills			
Very low confidence	0.0 (0)	0.0 (0)	0.0 (0)
Low confidence	0.0 (0)	1.1 (1)	0.8 (1)
Moderate confidence	21.2 (7)	27.0 (24)	25.4 (31)
High confidence	39.4 (13)	53.9 (48)	50.0 (61)
Complete confidence	39.4 (13)	18.0 (16)	23.8 (29)
Confidence in mental skills			
Very low confidence	0.0 (0)	2.2 (1)	1.6 (2)
Low confidence	0.0 (0)	3.4 (3)	2.5 (3)
Moderate confidence	33.3 (11)	33.7 (30)	33.6 (41)
High confidence	36.4 (12)	46.1(41)	43.4 (53)
Complete confidence	30.3 (10)	14.6 (13)	18.9 (23)
Confidence in technical skills-snatch			
Very low confidence	0.0 (0)	0.0 (0)	0.0 (0)
Low confidence	0.0 (0)	4.5 (4)	3.3 (4)
Moderate confidence	21.2 (7)	30.3 (27)	27.9 (34)
High confidence	63.6 (21)	48.3 (43)	52.5 (64)
Complete confidence	15.2 (5)	16.9 (15)	16.4 (20)
Confidence in technical skills-clean			
Very low confidence	0.0 (0)	0.0 (0)	0.0 (0)
Low confidence	3.0 (1)	3.4 (3)	3.3 (4)
Moderate confidence	24.2 (8)	18.0 (16)	19.7 (24)
High confidence	42.4 (14)	50.6 (45)	48.4 (59)
Complete confidence	30.3 (10)	28.1 (25)	28.7 (35)
Confidence in technical skills-jerk			
Very low confidence	0.0 (0)	0.0 (0)	0.0 (0)
Low confidence	3.0 (1)	2.2 (1)	2.5 (3)
Moderate confidence	42.4 (14)	25.8 (23)	30.3 (37)
High confidence	45.5 (15)	51.7 (46)	50.0 (61)
Complete confidence	9.1 (3)	20.2 (18)	17.2 (21)

**Table 5:** Exploratory factor analysis loadings for pre-competition confidence items. Loadings are from the retained three-factor solution representing Goals, Mental Resilience, and Physical Competition Readiness. Primary loadings are shown in bold. Items were assigned to factors based on the pattern of loadings and substantive interpretability.

Confidence Item	Factor 1 (Goals)	Factor 2 (Mental Resilience)	Factor 3 (Physical Competition Readiness)
Goal (snatch)	**0.687**	0.007	0.162
Goal (clean and jerk)	**0.940**	0.012	0.006
Goal (total)	**0.958**	−0.013	−0.006
Mental skills	0.010	**0.472**	0.276
Recovery after one missed lift	−0.038	**0.963**	0.016
Recovery after two missed lifts	0.026	**0.834**	−0.081
Technical skills (snatch)	−0.010	−0.127	**0.954**
Technical skills (clean)	0.032	0.309	**0.517**
Technical skills (jerk)	0.100	0.007	**0.614**
Physical skills	0.010	0.218	**0.710**
Training program	−0.092	**0.436**	**0.428**

Cross-loadings with absolute values ≥ |0.40| are shown in bold.

**Table 6:** Associations between pre-competition attentional cue categories and latent confidence constructs estimated using structural regression modeling (*n* = 122).

Predictor	*B*	SE	95% CI	*β*	*p*
F1: Goals (R^2^ = 0.085)					
Control	0.316	0.217	[−0.110, 0.742]	0.142	0.146
Neutral	0.352	0.310	[−0.254, 0.959]	0.119	0.255
Internal	−0.256	0.238	[−0.723, 0.211]	−0.103	0.282
External	0.406	0.224	[−0.034, 0.845]	0.188	0.071
Promotion	0.107	0.220	[−0.325, 0.538]	0.051	0.628
F2: Mental Resilience (R^2^ = 0.066)					
Control	0.455	0.225	[0.014, 0.896]	0.206	**0.043**
Neutral	−0.100	0.294	[−0.676, 0.477]	−0.034	0.735
Internal	−0.192	0.236	[−0.655, 0.271]	−0.078	0.417
External	0.021	0.221	[−0.413, 0.455]	0.010	0.925
Promotion	0.310	0.242	[−0.165, 0.786]	0.151	0.200
F3: Physical Competition Readiness (R^2^ = 0.082)					
Control	0.492	0.234	[0.033, 0.950]	0.221	**0.036**
Neutral	−0.125	0.314	[−0.741, 0.491]	−0.042	0.691
Internal	−0.005	0.222	[−0.440, 0.430]	−0.002	0.983
External	0.172	0.211	[−0.242, 0.586]	0.080	0.416
Promotion	0.435	0.221	[0.003, 0.868]	0.209	**0.049**

Note. *B* = unstandardized regression coefficient; SE = robust standard error; CI = confidence interval; *β* = standardized coefficient; *R^2^* = proportion of variance explained. All cue categories were entered simultaneously; factor covariances were freely estimated. The model was estimated using WLSMV with the 11 confidence items treated as ordered indicators. Bold *p*-values indicate statistical significance at *p* < 0.05.

## Data Availability

The survey instrument and cue-coding framework are available through the Open Science Framework repository (https://doi.org/10.17605/OSF.IO/GAKWZ). Individual participant data are not publicly available due to ethical and privacy restrictions. Summary results supporting the findings of this study are available within the article and Supplementary Materials.

## Supplementary Materials

The following supporting information can be downloaded at https://www.mdpi.com/article/10.3390/sports14100434/s1: Statistical methods, Table S1: Exploratory factor analysis model fit indices; Table S2: Confirmatory factor analysis results for the three-factor model of pre-competition confidence; Table S3: EFA and CFA factor correlations; Table S4: Estimated threshold for the confidence items.

## References

[1] Garhammer J. (1980). Power Production by Olympic Weightlifters. Med. Sci. Sports Exerc..

[2] Storey A., Smith H.K. (2012). Unique Aspects of Competitive Weightlifting: Performance, Training and Physiology. Sports Med..

[3] Moritz S.E., Feltz D.L., Fahrbach K.R., Mack D.E. (2000). The Relation of Self-Efficacy Measures to Sport Performance: A Meta-Analytic Review. Res. Q. Exerc. Sport.

[4] Bandura A. (1977). Self-Efficacy: Toward a Unifying Theory of Behavioral Change. Psychol. Rev..

[5] Jekauc D., Fiedler J., Wunsch K., Mülberger L., Burkart D., Kilgus A., Fritsch J. (2025). The Effect of Self-Confidence on Performance in Sports: A Meta-Analysis and Narrative Review. Int. Rev. Sport Exerc. Psychol..

[6] Vealey R.S. (1986). Conceptualization of Sport-Confidence and Competitive Orientation: Preliminary Investigation and Instrument Development. J. Sport Exerc. Psychol..

[7] Vealey R.S., Chase M.A. (2008). Self-Confidence in Sport. Advances in Sport Psychology.

[8] Lazarus R.S. (1999). Stress and Emotion: A New Synthesis.

[9] Nicholls A.R., Perry J.L., Calmeiro L. (2014). Precompetitive Achievement Goals, Stress Appraisals, Emotions, and Coping among Athletes. J. Sport Exerc. Psychol..

[10] Gencer E. (2021). Psychological Aspect of Pre-Competition Warm-up: Motivational Process and Outcomes. J. Educ. Issues.

[11] Neumann D.L. (2019). A Systematic Review of Attentional Focus Strategies in Weightlifting. Front. Sports Act. Living.

[12] Phylactou P. (2019). Inside the Mind of Weightlifters: The Mental Preparation of Greek-Cypriot Olympic-Weightlifting Athletes. J. Eur. Psychol. Stud..

[13] Porter J., Sims B. (2013). Altering Focus of Attention Influences Elite Athletes Sprinting Performance. Int. J. Coach. Sci..

[14] Schutts K.S., Wu W.F.W., Vidal A.D., Hiegel J., Becker J. (2017). Does Focus of Attention Improve Snatch Lift Kinematics?. J. Strength Cond. Res..

[15] Gottwald V., Davies M., Owen R. (2023). Every Story Has Two Sides: Evaluating Information Processing and Ecological Dynamics Perspectives of Focus of Attention in Skill Acquisition. Front. Sports Act. Living.

[16] Wulf G. (2013). Attentional Focus and Motor Learning: A Review of 15 Years. Int. Rev. Sport Exerc. Psychol..

[17] Memmert D. (2009). Pay Attention! A Review of Visual Attentional Expertise in Sport. Int. Rev. Sport Exerc. Psychol..

[18] Zhuravleva T.A., Aiken C.A. (2023). Adopting a Holistic Focus of Attention Promotes Adherence and Improves Performance in College Track and Field Athletes. Hum. Mov. Sci..

[19] Sadowski J., Szyszka P., Makaruk H., Starzak M., Niźnikowski T. (2024). The Effects of Holistic, External, and Internal Attentional Focus Instructions on Power and Kinematics of the Hang Power Snatch in Highly Skilled Weightlifters. J. Hum. Kinet..

[20] von Elm E., Altman D.G., Egger M., Pocock S.J., Gøtzsche P.C., Vandenbroucke J.P., STROBE Initiative (2008). The Strengthening the Reporting of Observational Studies in Epidemiology (STROBE) Statement: Guidelines for Reporting Observational Studies. J. Clin. Epidemiol..

[21] Anderson J.C., Gerbing D.W. (1988). Structural Equation Modeling in Practice: A Review and Recommended Two-Step Approach. Psychol. Bull..

[22] Mulaik S.A., Millsap R.E. (2000). Doing the Four-Step Right. Struct. Equ. Model. A Multidiscip. J..

[23] Horn J.L. (1965). A Rationale and Test for the Number of Factors in Factor Analysis. Psychometrika.

[24] Garrido L.E., Abad F.J., Ponsoda V. (2013). A new look at Horn’s parallel analysis with ordinal variables. Psychol. Methods.

[25] Hu L., Bentler P.M. (1999). Cutoff Criteria for Fit Indexes in Covariance Structure Analysis: Conventional Criteria versus New Alternatives. Struct. Equ. Model. Multidiscip. J..

[26] McDonald R.P. (1999). Test Theory: A Unified Treatment.

[27] Rosseel Y. (2012). Lavaan: An R Package for Structural Equation Modeling. J. Stat. Softw..

[28] Jorgensen T.D., Pornprasertmanit S., Schoemann A.M., Rosseel Y. (2012). semTools: Useful Tools for Structural Equation Modeling, version 0.5-8.

[29] Woodard K.F., Markwell L.T., Fairbrother J.T. (2021). Effects of an Expert-Modeled Attentional Focus Cue Structure on Skilled Jump Rope Performance and Learning. Hum. Mov. Sci..

[30] Liu G., Zhu H., Ma J., Pan H., Pan X., Zhang Y., Hu T., Fekete G., Guo H., Liang M. (2022). A Biomechanical Study on Failed Snatch Based on the Human and Bar Combination Barycenter. Appl. Bionics Biomech..

[31] Becker K.A., Georges A.F., Aiken C.A. (2019). Considering a Holistic Focus of Attention as an Alternative to an External Focus. J. Mot. Learn. Dev..

[32] Krenn B., Finkenzeller T., Würth S., Amesberger G. (2018). Sport Type Determines Differences in Executive Functions in Elite Athletes. Psychol. Sport Exerc..

[33] Chase M.A., Feltz D.L., Lirgg C.D. (2003). Sources of Collective and Individual Efficacy of Collegiate Athletes. Int. J. Sport Exerc. Psychol..

[34] Nagy L., Balázs K. (2023). Typical Coping Patterns: A Person-Centered Approach to Coping. New Ideas Psychol..

