# Supplementary material for: Verbal Cue Use and Domains of Pre-Competitive Confidence in Competitive Weightlifters
Source: Sports (Basel). 2026 Oct 6;14(10):434. doi: 10.3390/sports14100434 (PMC13709685; doi:10.3390/sports14100434)
Supplement: Supplementary file 1 [file sports-14-00434-s001.zip › sports-4549206-supplementary.pdf]

SUPPLEMENTARY MATERIALS

Exploratory Factor Analysis (EFA)

EFA Methods

To determine the latent structure of the confidence items, an exploratory factor analysis (EFA) was performed on the polychoric correlation matrix, which is appropriate for ordinal indicators. Two-, three-, and four-factor solutions were estimated using geomin rotation and the Unweighted Least Squares Mean and Variance adjusted (ULSMV) estimator. Geomin rotation was selected because the confidence domains were expected to be related rather than independent, and an oblique solution permits estimation of correlations among factors.
Factor retention was evaluated using multiple criteria, including model fit indices (CFI, TLI, RMSEA, and SRMR), parallel analysis, factor loading patterns and cross-loadings, improvement in fit across successive models, and substantive interpretability and parsimony.

- Model fit indices — CFI, TLI, RMSEA, and SRMR. Solutions were evaluated against conventional thresholds (CFI and TLI near or above 0.90; RMSEA and SRMR below approximately 0.08), with model fit judged collectively rather than by any single cutoff.
- Parallel analysis — conducted on the polychoric correlation matrix with 1,000 iterations (Horn, 1965; Garrido et al., 2013), comparing observed eigenvalues to those expected from random data.
- Factor loading patterns and cross-loadings — to assess whether each item loaded primarily on a single factor and whether the pattern was consistent with simple structure.
- Pattern of improvement across successive solutions — to determine whether adding a factor produced a meaningful gain in fit or only a marginal one.
- Substantive interpretability and parsimony — whether the resulting factors represented conceptually coherent and practically meaningful domains.

A solution was retained only if it (a) demonstrated acceptable overall fit, (b) produced a meaningful improvement over the preceding solution, (c) yielded conceptually coherent and interpretable factors, and (d) did not produce improper parameter estimates. For descriptive interpretation of the retained solution, loadings ≥ |0.40| were considered primary loadings, whereas loadings ≥ |0.30| on additional factors were considered meaningful cross-loadings. Items that did not exhibit simple structure were evaluated individually using both loading patterns and substantive interpretability.

EFA Results

Fit indices for the two-, three-, and four-factor solutions are presented in Table S1, and the scree plot and parallel analysis are shown in Figure S1. Parallel analysis suggested a two-factor solution. However, evaluation of model fit, loading patterns, substantive interpretability, and model admissibility supported retention of a three-factor solution. The two-factor solution combined conceptually distinct dimensions of confidence in recovering from missed lifts and

confidence in physical and technical readiness. The three-factor solution yielded interpretable factors representing Goals, Mental Resilience, and Physical Competition Readiness and demonstrated substantially improved fit. Although the four-factor solution produced slightly better fit statistics, the improvement over the three-factor solution was modest and the solution generated improper parameter estimates in sensitivity analyses. Accordingly, the three-factor solution was retained.

Table S1. Fit indices for candidate exploratory factor analysis solutions. Candidate two-, three-, and four-factor models were evaluated using ULSMV estimation with geomin rotation.

Factors	CFI	TLI	RMSEA	SRMR	ΔCFI	ΔRMSEA	Decision
2	0.970	0.955	0.075	0.056	—	—	Not retained
3	0.992	0.984	0.044	0.037	0.022	−0.031	**Retained**
4	0.997	0.991	0.032	0.028	0.005	−0.012	Not retained

Note. Δ values are relative to the preceding model.

The factors were moderately correlated (r = 0.492-0.631), supporting the use of an oblique rotation and indicating that the confidence domains were related but not redundant (Table S3).

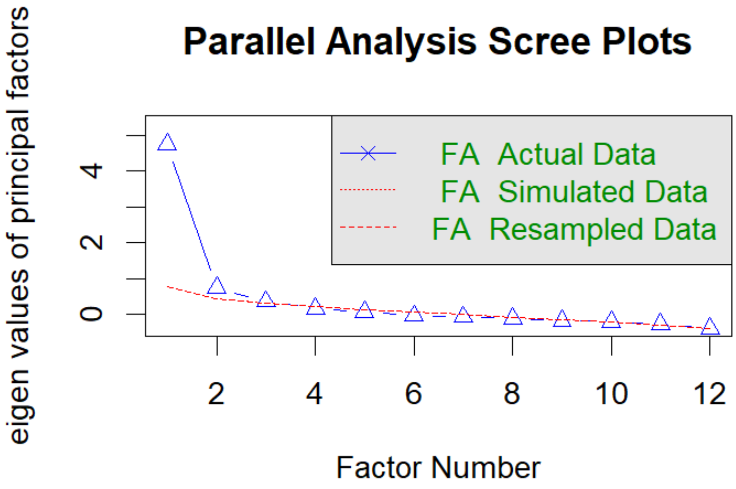

Figure S1. Scree plot and parallel analysis based on the polychoric correlation matrix. The solid line represents observed eigenvalues and the dashed line represents eigenvalues from randomly generated data (1,000 iterations).

The factor labels were assigned after examination of the item content and loading patterns and were not specified a priori. Factor 1 comprised confidence in achieving performance goals

(snatch, clean and jerk, and total) and was labeled *Goals*. Factor 2 was primarily characterized by confidence in recovering from missed lifts together with confidence in mental skills and was labeled *Mental Resilience*. Factor 3 comprised confidence in physical abilities, technical skills, and training preparation and was labeled *Physical Competition Readiness*. These labels were intended to reflect conceptually meaningful dimensions of confidence in competitive weightlifting.

Sensitivity analysis for the Training Program item.
Because the Training Program item demonstrated substantial cross-loadings on Mental Resilience and Physical Competition Readiness, the EFA was repeated after excluding this item. The resulting three-factor solution remained substantively unchanged, with comparable fit indices (CFI = 0.991 vs 0.992; RMSEA = 0.051 vs .044) and identical factor interpretation. Consequently, the item was retained because of its conceptual relevance to competition readiness and because its removal did not improve model performance.

Confirmatory Factor Analysis (CFA)

CFA Methods
The three-factor structure identified in the EFA was evaluated using confirmatory factor analysis (CFA). Because the confidence items were ordinal, all items were specified as ordered indicators (in the R lavaan package) and estimated using the weighted least squares mean- and variance-adjusted estimator (WLSMV). In *lavaan*, WLSMV uses diagonally weighted least squares (DWLS) parameter estimation together with robust standard errors and a mean- and variance-adjusted chi-square statistic. The model specified three correlated latent factors representing Goals**,** Mental Resilience, and Physical Competition Readiness**.** Model fit was evaluated using the scaled chi-square statistic, CFI, TLI, RMSEA with 90% confidence intervals, and SRMR. Reliability was assessed using McDonald's ω_{total}. Item communalities (h^2) and uniqueness values (u^2) were calculated from the standardized factor loadings, where $h^2 = \lambda^2$ represents the proportion of item variance explained by the latent factor and $u^2 = 1 - h^2$ represents unexplained variance, including measurement error. Consistent with commonly used SEM guidelines, CFI and TLI values near or above 0.90 and RMSEA and SRMR values below approximately 0.08 were used as descriptive benchmarks for acceptable fit. Because no single fit index is sufficient for model evaluation, model fit was assessed using the indices together with factor loading patterns, interpretability, and model admissibility rather than relying on strict cutoff criteria.

Four items (*confidence in training program*, *physical skills*, *the goal for the snatch*, and *goal for the clean & jerk*) contained fewer than five responses in the lowest category; therefore, the "Low confidence" and "Moderate confidence" categories were combined prior to analysis to ensure stable threshold estimation. The remaining items retained their original response categories. Models were estimated using the theta parameterization with factor variances fixed to one (*std.lv* = TRUE).

CFA Results

Model Fit. The chi-square statistic was significant ($\chi^2 = 75.309$, $p = .007$), indicating that exact model fit was not supported. However, the robust fit indices suggested that the model provided an acceptable representation of the data (CFI = 0.989, TLI = 0.986, RMSEA = 0.058, 90% CI [0.012, 0.090], SRMR = 0.057).

Factor Loadings and Construct Validity. Standardized factor loadings supported the three-factor structure (Table S2). Loadings for the Goals factor ranged from 0.839 to 0.973. The Mental Resilience factor was characterized by strong loadings for the recovery-after-missed-lift items (0.801-0.951), while the Mental Skills item demonstrated a more moderate loading (0.704). Standardized loadings for Physical Competition Readiness ranged from 0.670 to 0.867 across the technical skills, physical skills, and training preparation indicators. Item communalities ranged from 0.449 to 0.947, indicating that most items were well represented by their respective latent factors.

Table S2. Confirmatory factor analysis results for the three-factor model of pre-competition confidence (n = 122).

Factor / Item	Estimate	SE	p	Standardized (λ)	h^2	u^2
F1: Goals						
Snatch	0.828	0.052	< 0.001	0.839	0.704	0.296
Clean & Jerk	0.971	0.042	< 0.001	0.973	0.947	0.053
Total	0.908	0.041	<0.001	0.915	0.837	0.163
F2: Mental Resilience						
Mental skills	0.692	0.051	< 0.001	0.704	0.496	0.504
Recovery after one missed lift	0.947	0.041	< 0.001	0.951	0.904	0.096
Recovery after two missed lifts	0.791	0.045	< 0.001	0.801	0.642	0.358
F3: Physical Competition Readiness						
Technical skills (snatch)	0.773	0.046	< 0.001	0.786	0.618	0.382
Technical skills (clean)	0.791	0.048	< 0.001	0.803	0.645	0.355
Technical skills (jerk)	0.654	0.062	< 0.001	0.670	0.449	0.551
Physical skills	0.858	0.040	< 0.001	0.867	0.752	0.248
Training program	0.750	0.062	< 0.001	0.764	0.584	0.416

Note. Estimate = unstandardized factor loading; SE = standard error; Standardized (λ)= standardized factor loading; h^2 = communality (λ^2), the proportion of item variance explained by the latent factor; u^2 = uniqueness ($1 - h^2$), the proportion of item variance not explained by the factor (including measurement error). All loadings significant at $p < .001$.

Factor Correlations

The factors were moderately positively correlated in the EFA, with correlations ranging from 0.492 to 0.631 (Table S3), supporting the use of an oblique rotation. Correlations were somewhat higher in the CFA (0.673 to 0.746). This increase is expected because the CFA imposed a simple-structure model in which cross-loadings were fixed to zero, whereas the EFA allowed modest cross-loadings that absorbed some of the shared variance among factors. Despite these correlations, the factors remained conceptually distinct and represented different dimensions of pre-competition confidence.

Table S3. Correlations among factors from the retained three-factor exploratory and confirmatory factor analysis solution.

	EFA	CFA
Goals – Mental resilience	0.492	0.673
Goals – Physical Competition Readiness	0.631	0.746
Mental resilience – Physical Competition Readiness	0.561	0.590

Factor correlations were somewhat higher in the CFA than in the EFA. This pattern is expected because the EFA allowed modest cross-loadings, whereas the CFA imposed a more restrictive simple-structure model in which cross-loadings were fixed to zero.

Threshold estimates

Because the confidence items were modeled as ordered categorical indicators, threshold parameters were estimated as part of the WLSMV procedure (Table S4). Thresholds estimates for the ordinal confidence represent cut-points on the underlying latent response scale separating adjacent confidence categories.

Table S4. Estimated thresholds for the 11 ordinal confidence items (WLSMV, theta parameterization, $n = 122$).

Item (Factor)	Categories	τ_1 (SE)	τ_2 (SE)	τ_3 (SE)
**Goals**				
Goal (snatch)	4	−1.435 (0.302)	0.302 (0.258)	2.062 (0.452)
Goal (clean & jerk)	4	−1.406 (0.279)	0.450 (0.254)	2.024 (0.376)
Goal (total)	4	−0.942 (0.254)	0.738 (0.248)	2.241 (0.429)
**Mental Resilience**				
Mental skills	4	−1.644 (0.290)	−0.156 (0.224)	1.126 (0.242)
Recovery after one missed lift	4	−1.635 (0.305)	−0.236 (0.252)	1.382 (0.290)
Recovery after two missed lifts	4	−0.610 (0.254)	0.505 (0.261)	1.703 (0.328)
**Physical Competition Readiness**				
Training program	3	−0.696 (0.239)	0.759 (0.240)	—
Physical skills	3	−0.369 (0.250)	1.107 (0.264)	—
Technical skills (snatch)	3	−0.164 (0.249)	1.414 (0.287)	—
Technical skills (clean)	4	−1.425 (0.284)	−0.383 (0.228)	0.966 (0.233)
Technical skills (jerk)	3	0.093 (0.236)	1.504 (0.257)	—

Note. τ = threshold on the latent response scale; SE = standard error. Items with 4 categories have 3 thresholds; items with 3 categories have 2 thresholds.

References

Horn J. A rationale and test for the number of factors in factor analysis" Psychometrika 1965, **30** (2): *179*–185. doi:*10.1007/bf02289447*

Garrido LE, Abad FJ, Ponsoda V. A new look at Horn's parallel analysis with ordinal variables. Psychol Methods. 2013 Dec;18(4):454-74. doi: 10.1037/a0030005.
